# The Week's Work

**Published:** 1909-10-16

**Authors:** 


### The Week's Work.

Sir Henry Burdett's report on the North Staffordshire Infirmary has created a good deal of interest in the large district which this hospital serves. Mr. W. Guy Knight, the President of the Infirmary, has written a letter to the local papers in which he points out the important character of the report, which is so disquieting to the many generous friends of the Institution that the Committee desires to place a statement on the subject before the public. Mr. Knight proceeds to say that the report confines itself almost entirely to the defects and ignores the merits of the Infirmary, and the numerous alterations and improvements of recent years. These improvements comprise the reorganisation of the drainage, the erection of an isolation block, the installation of electric light, the better sterilising of dressings, alterations to the theatre and its adjuncts, the reorganisation of the mortuary and post-mortem rooms, and new lavatories for the large wards, involving a total cost of about £5,000. It is a little disturbing as a test of the knowledge and capacity of the present managers of the Infirmary that Mr. Knight should claim credit for the Isolation Block and the money spent upon it, seeing that further on he mentions that the Committee contemplates its improvement. Sir Henry indeed reported that the Isolation Block " is a temporary iron structure, the sanitation, heating and comfort of which are deplorable. It would be cruel to think of putting any patient on any pretence into these wards as they exist to-day."

Mr. Knight next declares that the main criticisms of the report refer to the out-patient department, the patients treated having increased in ten years from about 10,000 to 14,000. The Committee fully recognises that the accommodation is inadequate, and a sub-Committee is engaged on a scheme for its reconstruction at a cost of £3,000. He states that further schemes comprising many of the points raised in the report are in contemplation. The President holds that the £60,000 invested can only in part be applied to these works, and the Committee would hesitate to disturb any part of them for the purpose. The question, the President maintains, is one of finance, and if the district will find sufficient funds the Committee will make the North Staffordshire Infirmary as perfect as possible. With this object in view it is proposed shortly to issue an appeal to the public for a large sum of money.

It is obvious from the President's letter that neither he nor his colleagues have that intimate and close knowledge of the working of the Institution and the management of its affairs which is essential to the efficient administration of a great public hospital. The President ignores altogether the important facts (1) that an immediate increase of the honorary medical staff by the addition of at least two more surgeons and physicians is urgently called for; (2) that at the present time the Hospital is understaffed, having regard to the volume of medical and surgical work which the number of both in- and out-patients involves; (3) that the past history and present condition of the Infirmary must convince every intelligent governor, who complies with Sir Henry's suggestion to inspect the Infirmary on a busy day and to judge for himself, that the present state of affairs is so serious that it is essential that the governors should appoint a Special Committee with power to employ expert advice, and to bring up a report comprising all the points at issue, including the revision of the present rules and regulations, especially in regard to the number, qualifications and duties of the honorary medical staff. We take it that those governors who carefully study Mr. Guy Knight's letter must realise that the relatively small matters which have been attempted by the Committee in recent years, and the condemnation of its administration which the present Isolation Block supplies, make it imperative that they should at their annual meeting select from their body a few of the most intelligent and able of their number, and entrust them with the duty of making this enquiry, with the object of securing that no money shall be spent upon buildings or reconstruction for the future until evidence is forthcoming, that, the proposed changes will produce the results desired.

The Potteries comprise a large district, and the North Staffordshire Infirmary has to minister to the needs of a population which approaches, if it does not exceed, 400,000 people. This population has shown a notable spirit of thrift, and the working men hand each year to the Infirmary authorities a sum which is practically equivalent to half its annual income. According to the last accounts available the artisans contributed by establishment subscriptions £5,705 out of a total ordinary income of £12,520. Pecuniary support of this generous nature makes the claim of the working classes upon the management paramount. We hope that these workmen governors may have sufficient public spirit to make it their business to confer, together, so that their representatives may be fully instructed as to the policy they will pursue at the annual meeting of governors, which will be held, we understand, in November. We have confidence that the governors at that meeting will insist upon adequate steps being taken through the instrumentality of a Special Committee to ascertain the actual state and needs of the North Staffordshire Infirmary. With the aid of expert advice this Special Committee should be able to prepare a scheme for the consideration of an adjourned meeting of governors to be held within a reasonable time. If this is done, and the medical staff is strengthened by the appointment of additional physicians and surgeons of the first rank, confidence will be restored, and the requirements of this large district, so far as hospital needs are concerned, will be adequately met.

Our contemporary *The Stafforshire Sentinel* is rendering good service to the public by encouraging a discussion on Sir Henry Burdett's report on the North Staffordshire Infirmary and devoting a good deal of space to the matter. In a leading article *The Sentinel*, however, misapprehends some points of importance. Sir Henry had written 50 reports upon the hospitals he had inspected before he arrived at Stoke, but his report on the North Staffordshire Infirmary was published first because the annual meeting of that institution is due to be held in November. Six weeks is none too long a period to enable the governors to organise themselves and to prepare for a full discussion and a large attendance at the annual meeting. We publish in another column an extract from *Truth*, which disposes of the error made by the *Sentinel* in regard to Sir Henry's reports. They will duly be published, and should constitute as interesting a book of reference as the reports of Mr. Holmes and Dr. Bristowe made in 1863. Our contemporary fails to grasp that the position of the North Staffordshire Infirmary to-day points to an absence of continuous oversight and interest on the part of the managing committee and honorary staff. There is a weekly committee, we understand, but the names of those who constitute it do not appear in the annual report. It is desirable that the number of members and the attendance at each weekly committee for the last twelve months should be made known to the governors. These figures might help to explain why matters have been allowed to drift into their present condition.

THE *Sentinel* has evidently no experience of the actual management and working of a great hospital, or it would not commit itself in a leading article to the statement that the increase of the medical staff is a matter for the present members of that staff in the first instance to determine. The infirmary contains 216 beds, but the medical staff at present consists of only two physicians and two assistant physicians, with two surgeons and two assistant surgeons, in addition to the ophthalmic and special departments and dental staff. It will be seen, therefore, that two additional physicians and two additional surgeons, at the least, are required to do the work, and bring the number of the staff to the level of efficiency attained by other similar institutions. This point should be very carefully gone into by a special committee of governors, for it lies at the root of the present mischief and calls for the promptest action on their part. This increase of the staff is a matter of urgent business that must not be neglected or allowed to drop. The *Sentinel* and Mr. Guy Knight are both under a misapprehension in regard to the suggestion made in relation to the £60,000 invested. That suggestion was not that this money should be sunk in buildings or alterations, but that the relatively small proportion of it required to do what is necessary might be temporarily taken from this fund, pending the raising of the money from the public, so as to secure that immediate steps shall be taken to bring the whole infirmary up to date in the best interests of everybody concerned.

WE would call attention to an interesting communication from the Hon. Sydney Holland on an eight hours' day for nurses. There can be no doubt that in too many hospitals the hours on duty for nurses are too long. A working day of 13 hours, with only two hours off duty, is much longer than it ought to be. Indeed, we find that wherever the nursing department is under the control of an able and conscientious matron there these hours are modified, or a continuous effort is sustained with a view to securing their modification. Mr. Holland states his reasons, based upon experience, why in his opinion it is not only impossible, but undesirable, to have an eight hours' day for nurses. We should be glad to have further views on this question and to publish them in due course. It concerns members of the medical profession rather closely, especially if they are surgeons, for a nurse who is overtired is not in a condition to be placed in sole charge of a serious operation case. This is now so generally recognised that some hospitals keep a few extra nurses that they may be available for such cases when necessary.

OF late years an increasing interest has been displayed in regard to fire appliances and fire escapes in connection with public buildings, and especially with hospitals. Articles on these subjects appeared in THE HOSPITAL on May 29 and June 5 last. We welcome this week an inquiry on the subject from a member of the Committee of the Bolton Infirmary, whose questions appear on another page. We hope that any committeeman who has any point of difficulty upon which he seeks information or guidance will follow Mr. Kay's example and communicate with us. It would be difficult to overestimate the amount of correspondence and postage which might be saved if this course were generally followed by all connected with the voluntary hospitals. For then a file of THE HOSPITAL would soon constitute a boow of reference to which all hospital workers might apply whenever they found themselves in a difficulty. On the greater questions of construction and administration many hospital administrators at present find the file of THE HOSPITAL most useful; but we hope with the aid of our readers to extend this usefulness by giving space to questions and answers on every and any point which may have an interest for hospital workers. That is why we welcome cordially the communication of Mr. Kay, and commend his example to members of hospital committees generally.

THE article published last week, giving the operation theatre unit, is one to be studied and criticised. We noticed this week, in inspecting a relatively new theatre from the plans of Mr. Percy Adams at Tunbridge Wells, that the only provision for cleaning instruments and washing up after each operation were the sinks in the theatre itself. It must be most inconvenient and objectionable for a surgeon to have to operate amidst the noise and discomforts of scullery operations. Hygienically such an arrangement has nothing to support it, but quite the contrary. We believe a very useful purpose might be served if the Royal Society of Medicine, in conjunction with the Royal Institute of British Architects, were to appoint a small, carefully selected committees to prepare model units for each department of a modern hospital. At the present time the more closely plans of new hospitals are studied the more clear it becomes that practical points are continually overlooked.

